# A chromosomal reference genome sequence for the malaria mosquito, *Anopheles marshallii*, Theobald, 1903

**DOI:** 10.12688/wellcomeopenres.22989.1

**Published:** 2024-09-26

**Authors:** Boris K. Makanga, Diego Ayala, Nil Rahola, Lemonde B. A. Bouafou, Harriet F Johnson, Haynes Heaton, Martin G. Wagah, Joanna C. Collins, Ksenia Krasheninnikova, Sarah E. Pelan, Damon-Lee B. Pointon, Ying Sims, James W. Torrance, Alan Tracey, Marcela Uliano-Silva, Jonathan M. D. Wood, Katharina von Wyschetzki, Wellcome Sanger Institute Scientific Operations: Sequencing Operations, Shane A. McCarthy, Daniel E. Neafsey, Alex Makunin, Mara K. N. Lawniczak

**Affiliations:** 1 Département de Biologie et Écologie Animale, Institut de Recherche en Écologie Tropicale, Libreville, Gabon; 2 MIVEGEC, University of Montpellier, CNRS, IRD, Montpellier, France; 3 Medical Entomology Unit, Institut Pasteur de Madagascar, Antananarivo, Antananarivo Province, Madagascar; 4 ESV, Centre Interdisciplinaire de Recherches Médicales de Franceville (CIRMF), Franceville, Gabon; 5 Scientific Operations, Wellcome Sanger Institute, Hinxton, England, UK; 6 CSSE, Auburn University, Auburn, Alabama, USA; 7 Tree of Life, Wellcome Sanger Institute, Hinxton, England, UK; 8 Department of Genetics, University of Cambridge, Cambridge, England, UK; 9 Department of Immunology and Infectious Diseases, Harvard T.H. Chan School of Public Health, Boston, MA, USA; 10 Infectious Disease and Microbiome Program, Broad Institute, Cambridge, Massachusetts, USA

**Keywords:** Anopheles marshallii, African malaria mosquito, genome sequence, chromosomal

## Abstract

We present a genome assembly from an individual female *Anopheles marshallii* (the malaria mosquito; Arthropoda; Insecta; Diptera; Culicidae) from Lopé, Gabon. The genome sequence is 225.7 megabases in span. Most of the assembly is scaffolded into three chromosomal pseudomolecules with the X sex chromosome assembled. The complete mitochondrial genome was also assembled and is 15.4 kilobases in length.

## Species taxonomy

Animalia; Arthropoda; Insecta; Diptera; Culicidae; Anophelinae; Anopheles; *Anopheles marshallii*; Theobald, 1903 (NCBI txid:1521116).

## Background

*Anopheles marshallii* (Theobald, 1903) is a secondary malaria vector that inhabits across sub-Saharan Africa ^1, 2^. Together with twelve other species, *An. austenii*, *An. berghei*, *An. brohieri*, *An. hancocki*, *An. hargreavesi*, *An. mortiauxi*, *An. mousinhoi*, *An. njombiensis*, *An. seydeli*, *An. hughi*, *An. kosiensis* and *An. letabensis*, it forms the Marshallii Group ^3^. The last three species constitute the Marshallii complex with *An. marshallii*. This mosquito is present almost everywhere on the continent, from West Africa (Sierra Leone, Cote d’Ivoire, Ghana, etc) to Central Africa (Cameroon, Gabon, Congo, etc), East Africa (Ethiopia, Kenya, Tanzania, etc) and in a few Southern African countries such as Angola, Burundi and South Africa ^4– 6^.

*An. marshallii* larvae are usually present in streams and swampy areas, where the water is relatively clear and vegetation is abundant ^7– 9^. *An, marshallii* had been considered medically unimportant because of its zoophilic behaviour, but over time its generalist, including feeding on humans, behaviour and vectorial ability were demonstrated in the laboratory and the field ^1, 2^. It has been found in rural areas biting humans ^10^, but also in the tropical forests of central Africa far from the presence of humans ^11^, where it is involved in the transmission of non-human primates malaria ^11, 12^. The generalist behaviour of this mosquito also makes it one of the potential bridge vectors in the transfer of malaria from non-human primates to humans ^12^. In addition, *An. marshallii* is capable of feeding on a wide range of hosts (rodents, wild ungulates, great apes, and bats) and is one of the vectors of the parasites described in this wildlife ^13, 14^.

Most of the genetic work done on *An. marshallii* has focused on the detection of cryptic species within the Marshallii complex ^15^. To this end, chromosomal or isoenzymatic markers were used for the identification of four cryptic species ^16– 18^. In recent years, the use of molecular markers based on sequence variation within the ITS1 region or the mitochondrial genes (COI, COII) have made it possible to distinguish species within the group ^19– 21^. However, these genetic markers have not been involved in population genetic studies, neither to characterise the more or less zoophilic populations.

The genome of the African malaria mosquito, *Anopheles marshallii*, was sequenced as part of the Anopheles Reference Genomes Project (PRJEB51690). Here we present a chromosomally complete genome sequence for *Anopheles marshallii*, based on a single female from Lopé, Gabon.

## Genome sequence report

The genome was sequenced from a single female *Anopheles marshallii* reared from a female mosquito collected in Lopé, Gabon (-0.187, 11.611) in April 2019. A total of 51-fold coverage in Pacific Biosciences single-molecule HiFi long reads (N50 7.850 kb) and 86-fold coverage in 10X Genomics read clouds were generated. Primary assembly contigs were scaffolded with chromosome conformation Hi-C data from a sibling female individual.

**Figure 1.**
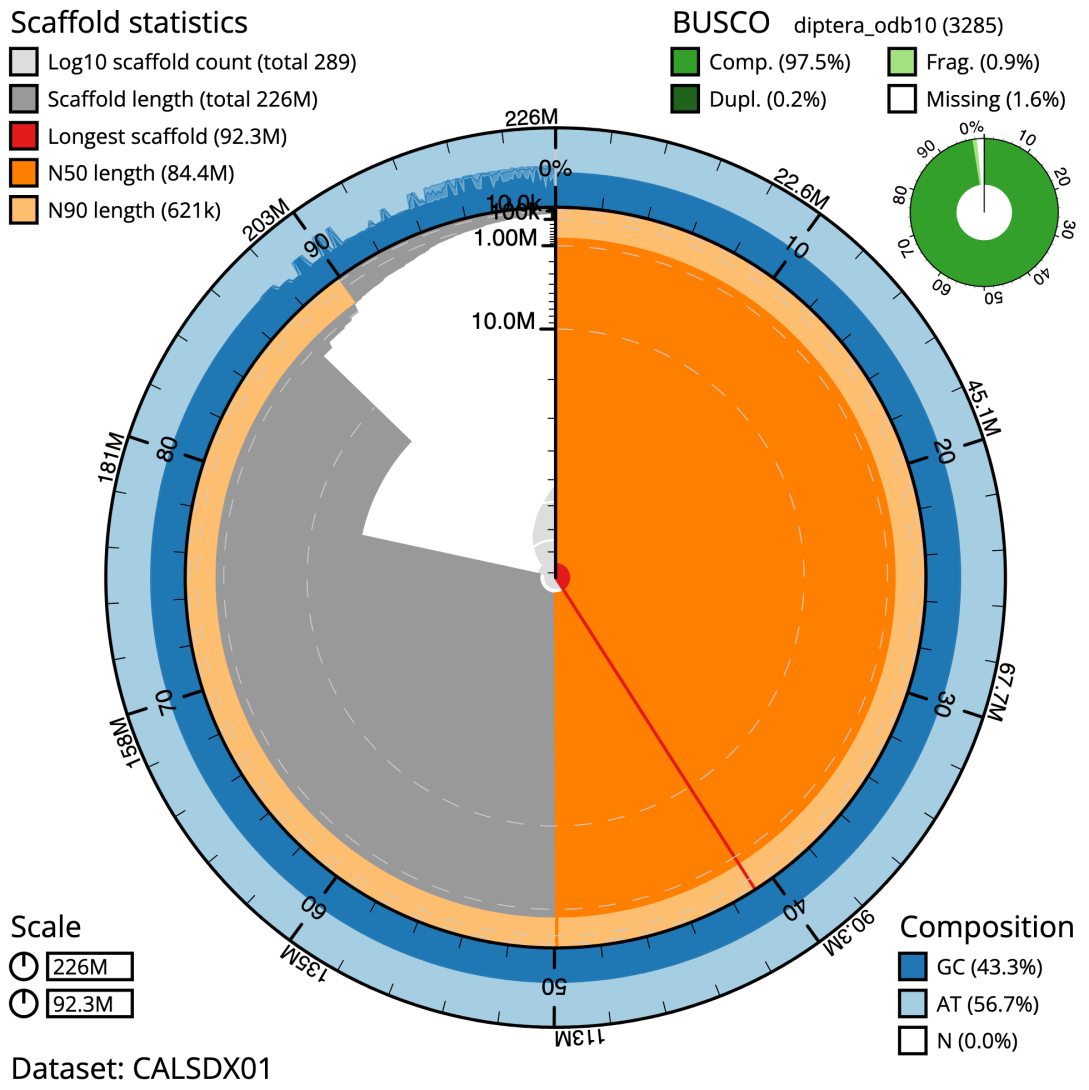
Snail plot summary of assembly statistics for *Anopheles marshallii* assembly. The main plot is divided into 1,000 size-ordered bins around the circumference with each bin representing 0.1% of the 225,727,465 bp assembly. The distribution of sequence lengths is shown in dark grey with the plot radius scaled to the longest sequence present in the assembly (92,345,055 bp, shown in red). . Orange and pale-orange arcs show the N50 and N90 sequence lengths (84,416,232 and 620,820 bp), respectively. The pale grey spiral shows the cumulative sequence count on a log scale with white scale lines showing successive orders of magnitude. The blue and pale-blue area around the outside of the plot shows the distribution of GC, AT and N percentages in the same bins as the inner plot. A summary of complete, fragmented, duplicated and missing BUSCO genes in the diptera_odb10 set is shown in the top right. An interactive version of this figure is available at https://blobtoolkit.genomehubs.org/view/idAnoMarsDA_429_01/dataset/CALSDX01/snail.

**Figure 2.**
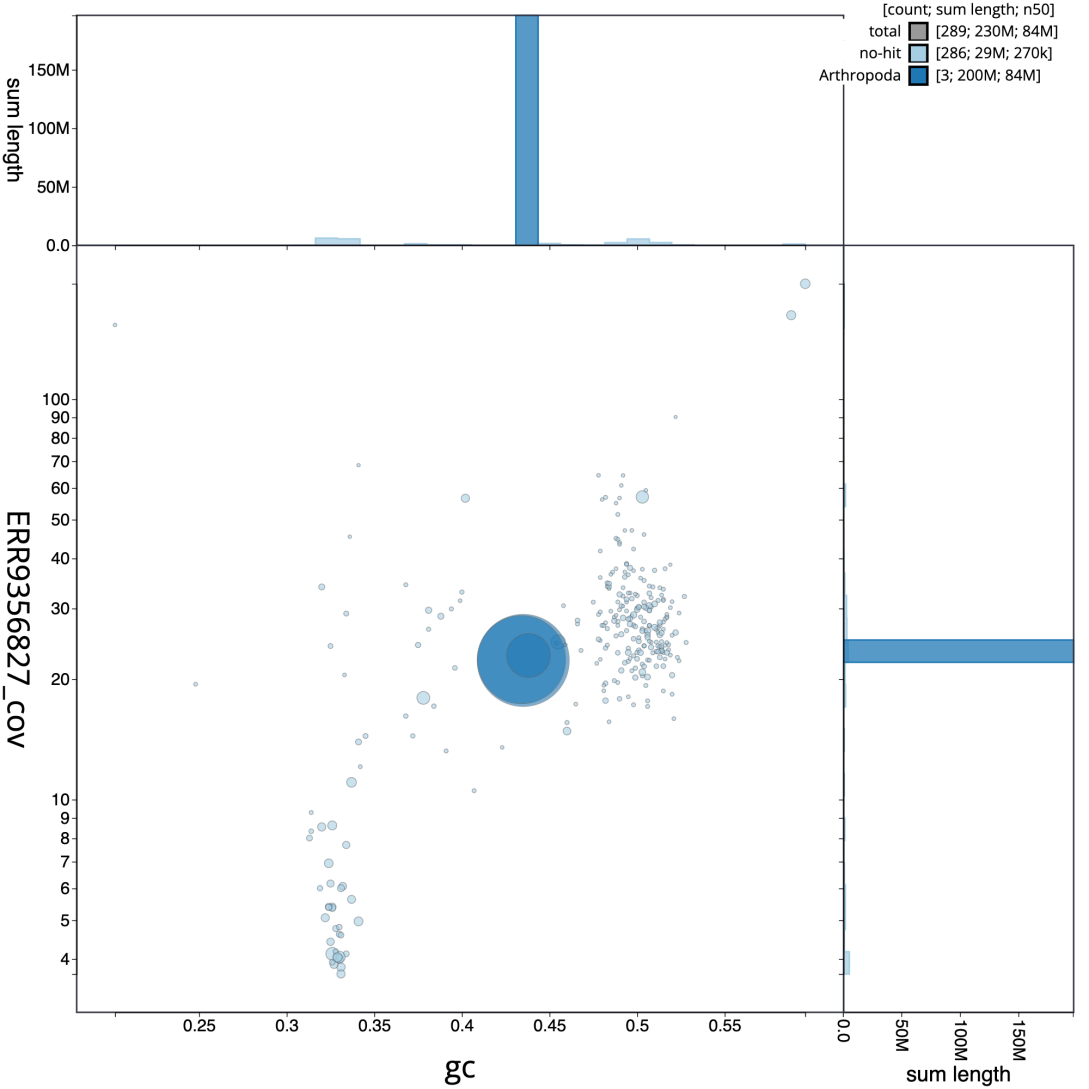
Blob plot of base coverage in a subset of idAnoMarsDA-429_01 10x linked reads against GC proportion for *An. marshallii* assembly idAnoMarsDA_429_01 Chromosomes are coloured by phylum. Circles are sized in proportion to chromosome length. Histograms show the distribution of chromosome length sum along each axis. An interactive version of this figure is available at https://blobtoolkit.genomehubs.org/view/idAnoMarsDA_429_01/dataset/CALSDX01/blob.

**Figure 3.**
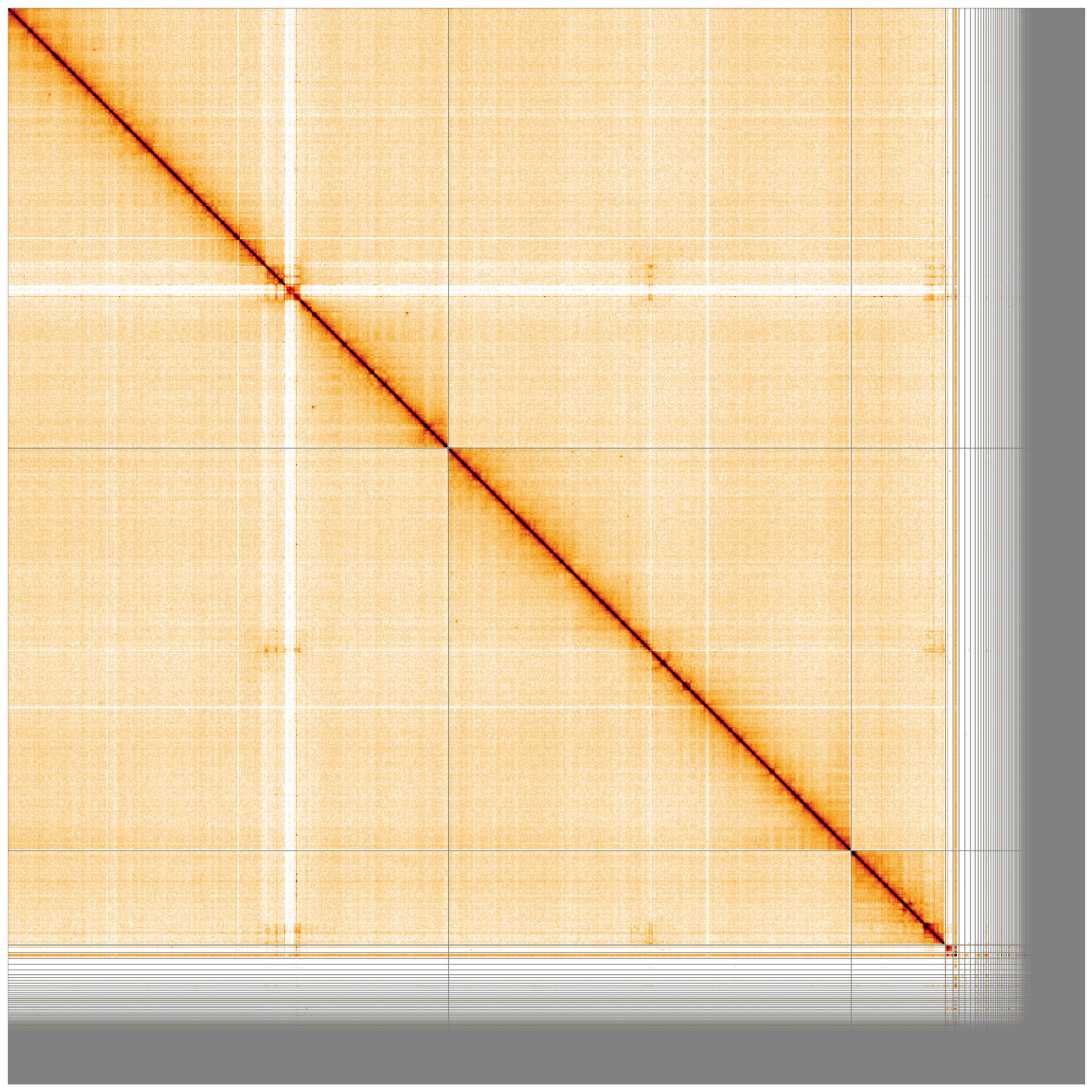
Genome assembly of *An. marshallii*, idAnoMarsDA_429_01: Hi-C contact map. Visualised in HiGlass. Chromosomes order: 2RL, 3RL, X, followed by unlocalised scaffolds. The interactive Hi-C map can be viewed at https://genome-note-higlass.tol.sanger.ac.uk/l/?d=PVHgSVbbST-xI-BNy6pPIA

**Figure 4.**
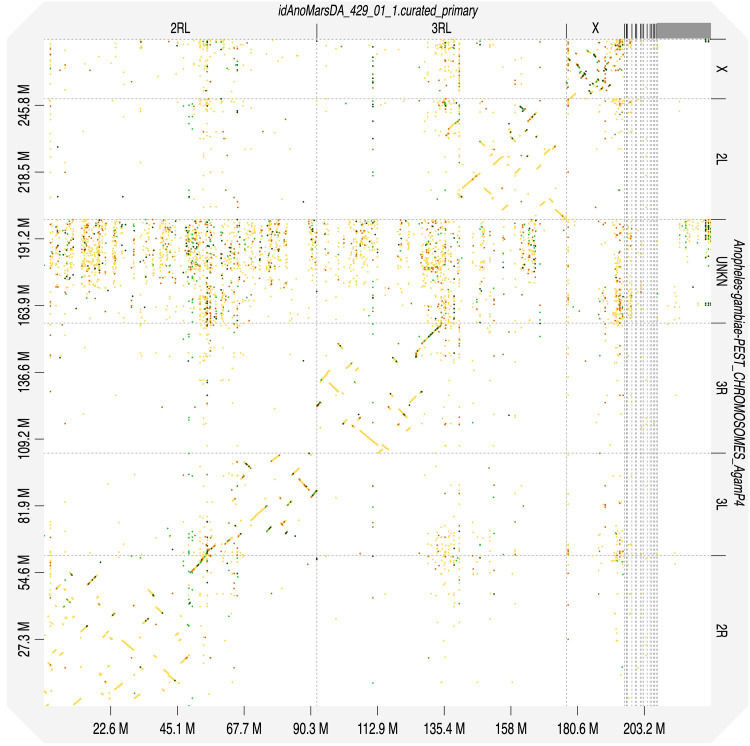
Alignment dotplot between genome assemblies of *An. marshallii,* idAnoMarsDA_429_01 and *An. gambiae*, AgamP3 (PEST). Chromosome arms correspondence (marshallii-gambiae): 2R-2R, 2L-3L, 3R-3R, 3L-2L in agreement with 23.

The final assembly has a total length of 225.7 Mb in 289 sequence scaffolds with a scaffold N50 of 84.416 Mb ( Table 1). The snail plot in Figure 1 provides a summary of the assembly statistics, while the distribution of assembly scaffolds on GC proportion and coverage is shown in Figure 2. The assembly sequence was assigned to three chromosomal-level scaffolds, representing two autosomes and the X sex chromosome ( Figure 3; Table 2). Chromosomes were numbered and oriented using synteny to the AgamP3 assembly ( 22; GCF_000005575.2) based on cytogenetics data for other Myzomyia series species ^23^) ( Figure 4). The assembly has a BUSCO 5.3.2 ^24^ completeness of 97.5% using the diptera_odb10 reference set. While not fully phased, the assembly deposited is of one haplotype and also includes the circular mitochondrial genome. Contigs corresponding to the second haplotype have also been deposited.

**Table 1.** Genome data for *An. marshallii,* idAnoMarsDA_429_01.

*Project accession data*		
Assembly identifier	idAnoMarsDA_429_01	
Species	*Anopheles marshallii*	
Specimen	idAnoMarsDA-429_01	
NCBI taxonomy ID	1521116	
BioProject	PRJEB53264	
BioSample ID	ERS10527370	
Isolate information	female, whole organism	
*Raw data accessions*		
PacificBiosciences SEQUEL II	ERR9439506	
10X Genomics Illumina	ERR9356824, ERR9356825, ERR9356826, ERR9356827	
Hi-C Illumina	ERR9356819	
PolyA RNA-Seq Illumina	ERR9356828, ERR9356829	
*Genome assembly*		
Assembly accession	GCF_943734725	
Accession of alternate haplotype	GCA_943734855	
Span (Mb)	225.727	
Number of contigs	305	
Contig N50 length (Mb)	32.607	
Number of scaffolds	289	
Scaffold N50 length (Mb)	84.416	
Longest scaffold (Mb)	92.345	
BUSCO * genome score	C:97.5%[S:97.3%,D:0.2%],F:0.9%,M:1.6%,n:3,285	

* BUSCO scores based on the diptera_odb10 BUSCO set using BUSCO 5.3.2. C=complete [S=single copy, D=duplicated], F=fragmented, M=missing, n=number of orthologues in comparison. A full set of BUSCO scores is available at https://blobtoolkit.genomehubs.org/view/idAnoMarsDA_429_01/dataset/CALSDX01/busco.

**Table 2.** Chromosomal pseudomolecules in the genome assembly of *An. marshallii,* idAnoMarsDA_429_01.

INSDC accession	Chromosome	Size (Mb)	Count	Gaps
**OX030903.1**	2RL	92.345	1	6
**OX030904.1**	3RL	84.416	1	3
**OX030905.1**	X	19.747	1	1
**OX030906.1**	MT	0.015	1	0
	X Unlocalised	29.203	285	6

**Figure 5.**
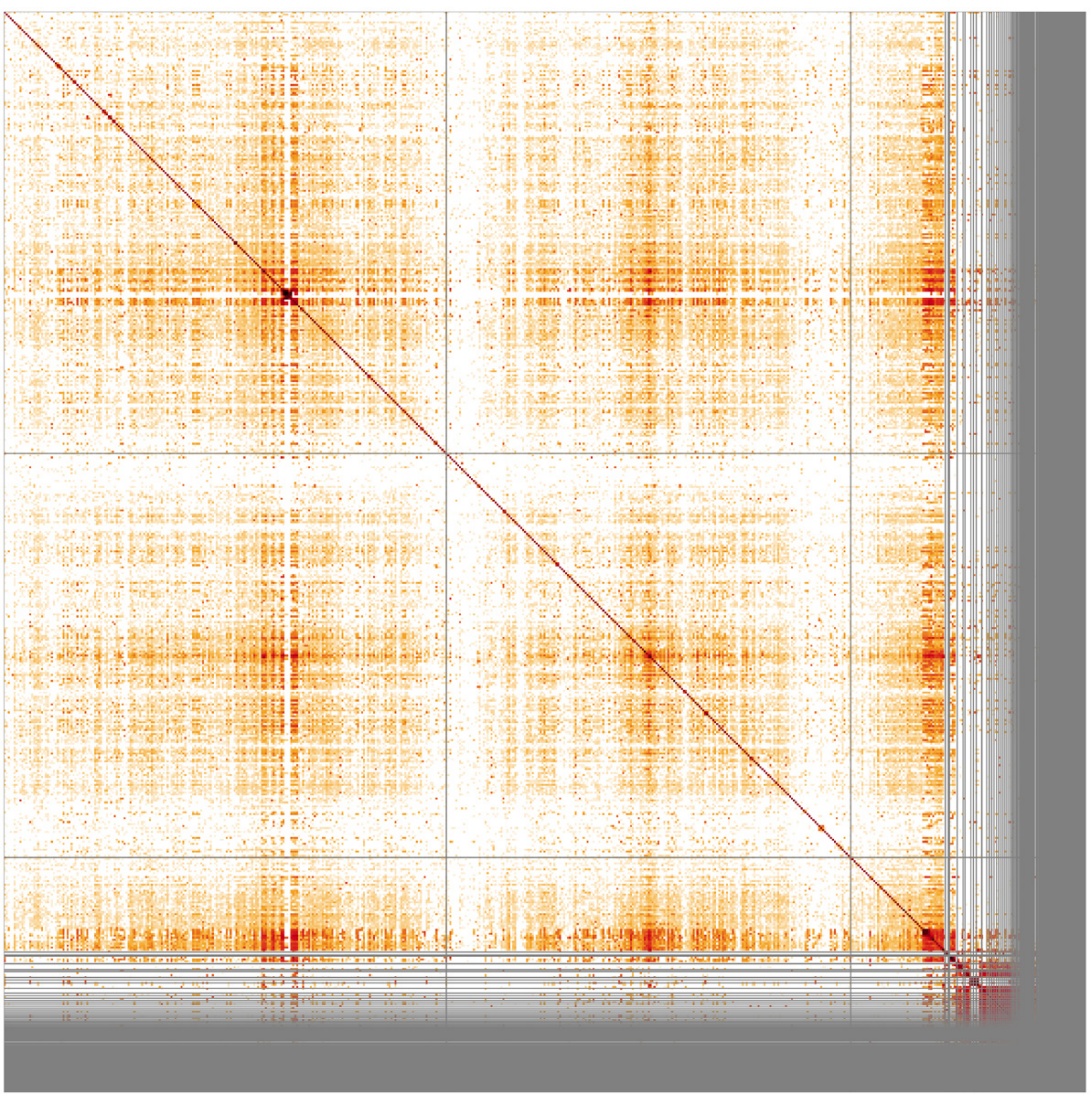
Sequence similarity heatmap for genome assembly of *An. marshallii*, idAnoMarsDA_429_01. Produced with StainedGlass, visualised in HiGlass. Chromosomes order: 2RL, 3RL, X - followed by unlocalised scaffolds. Darker colours represent higher sequence similarity, notably at pericentric and intercalary heterochromatin as well as in unassembled scaffolds.

Chromosome arms, candidate centromere sequences, and the rDNA region were delineated based on the presence of characteristic tandem repeat arrays ( Figure 5; Table 3). Candidate centromere regions in 2RL, 3RL and X comprised 189bp tandem repeat blocks. Predicted centromere locations agree well with synteny to *An. gambiae* ( Figure 4) and Hi-C data ( Figure 3). Clusters of rRNA genes were found on multiple unlocalised X-linked contigs.

**Table 3.** Chromosome arms in the genome assembly of *An. marshallii,* idAnoMarsDA_429_01.

Chromosome	Start	End	Chromosome arm
**2RL**	1	54,056,034	2R
**2RL**	54,238,357	92,345,055	2L
**3RL**	1	42,547,211	3R
**3RL**	42,764,533	84,416,232	3L
**X**	1	19,545,021	X

Gene annotation was performed with the NCBI Eukaryotic Genome Annotation Pipeline and is available in RefSeq ^25^ under the accession GCF_943734725.1. A total of 13,358 genes were predicted, including 11,720 protein-coding genes and 1,204 non-coding RNAs. The genome assembly and gene annotations are hosted on VectorBase, www.vectorbase.org ^26^ under the identifier AmarGA1.

## Methods

### Sample acquisition and nucleic acid extraction

*Anopheles marshallii* offspring were reared from a wild caught gravid female collected outdoors using human landing catch. A single female offspring idAnoMarsDA-429_01 was used for Pacific BioSciences and 10x genomics, a sibling female idAnoMarsDA-429_03 was used for Arima Hi-C.

For high molecular weight (HMW) DNA extraction one whole insect (idAnoMarsDA-429_01) was disrupted by manual grinding with a blue plastic pestle in Qiagen MagAttract lysis buffer and then extracted using the Qiagen MagAttract HMW DNA extraction kit with two minor modifications ^27^. The quality of the DNA was evaluated using an Agilent FemtoPulse to ensure that most DNA molecules were larger than 30 kb, and preferably > 100 kb. In general, single mosquito extractions ranged in total estimated DNA yield from 200 ng to 800 ng, with an average yield of 500 ng. Low molecular weight DNA was removed using an 0.8X AMpure XP purification. A small aliquot (less than ~5% of the total volume) of HMW DNA was set aside for 10X Linked Read sequencing and the rest of the DNA was sheared to an average fragment size of 12-20 Kb using a Diagenode Megaruptor 3 at speeds ranging from 27 to 30.Sheared DNA was purified using AMPure PB beads with a 1.8X ratio of beads to sample to remove the shorter fragments and concentrate the DNA sample. The concentration and quality of the sheared and purified DNA was assessed using a Nanodrop spectrophotometer and Qubit Fluorometer with the Qubit dsDNA High Sensitivity Assay kit. Fragment size distribution was evaluated by running the sheared and cleaned sample on the FemtoPulse system once more. The median DNA fragment size for *Anopheles* mosquitoes was 15 kb and the median yield of sheared DNA was 200 ng, with samples typically losing about 50% of the original estimated DNA quantity through the process of shearing and purification.

For Hi-C data generation, a separate sibling mosquito specimen (idAnoMarsDA-429_03) was used as input material for the Arima V2 Kit according to the manufacturer’s instructions for animal tissue. This approach of using a sibling was taken in order to enable all material from a single specimen to contribute to the PacBio data generation given we were not always able to meet the minimum suggested guidance of starting with > 300 ng of HMW DNA from a specimen. Samples proceeded to the Illumina library prep stage even if they were suboptimal (too little tissue) going into the Arima reaction.

To assist with annotation, which will be made available through VectorBase ^26^ in due course, RNA was extracted from separate whole lab-reared offspring reared from an unrelated wild-caught female mosquito (female idAnoMarsDA-426_03 and male idAnoMarsDA-426_10) using TRIzol, according to the manufacturer’s instructions. RNA was then eluted in 50 μl RNAse-free water, and its concentration was assessed using a Nanodrop spectrophotometer and Qubit Fluorometer using the Qubit RNA Broad-Range (BR) Assay kit. Analysis of the integrity of the RNA was done using Agilent RNA 6000 Pico Kit and Eukaryotic Total RNA assay. Samples were not always ideally preserved for RNA, so qualities varied but all were sequenced anyway.

### Sequencing

We prepared libraries as per the PacBio procedure and checklist for SMRTbell Libraries using Express TPK 2.0 with low DNA input. Every library was barcoded to support multiplexing. Final library yields ranged from 20 ng to 100 ng, representing only about 25% of the input sheared DNA. Libraries from two specimens were typically multiplexed on a single 8M SMRT Cell. Sequencing complexes were made using Sequencing Primer v4 and DNA Polymerase v2.0. Sequencing was carried out on the Sequel II system with 24-hour run time and 2-hour pre-extension. A 10X Genomics Chromium read cloud sequencing library was also constructed according to the manufacturer’s instructions (this product is no longer available). Only 0.5 ng of DNA was used and only 25–50% of the gel emulsion was put forward for library prep due to the small genome size. For Hi-C data generation, following the Arima HiC V2 reaction, samples were processed through Library Preparation using a NEB Next Ultra II DNA Library Prep Kit and sequenced aiming for 100x depth. RNA libraries were created using the directional NEB Ultra II stranded kit. Sequencing was performed by the Scientific Operations core at the Wellcome Sanger Institute on Pacific Biosciences SEQUEL II (HiFi), Illumina NovaSeq 6000 (10X and Hi-C), or Illumina HiSeq 4000 (RNAseq).

### Genome assembly

Assembly was carried out with Hifiasm ^28^; haplotypic duplications were identified and removed with purge_dups ^29^. One round of polishing was performed by aligning 10X Genomics read data to the assembly with Long Ranger ALIGN, calling variants with FreeBayes ^30^. The assembly was then scaffolded with Hi-C data ^31^ using SALSA2 ^32^. The assembly was checked for contamination as described previously ^33^. Manual curation was performed using gEVAL ^34^, HiGlass ^35^ and Pretext ^36^. The mitochondrial genome was assembled using MitoHiFi ^37^, which performs annotation using MitoFinder ^38^. The genome was analysed and BUSCO scores were generated within the BlobToolKit environment ^39^. Synteny analysis was performed with D-GENIES ^40^ and minimap2 ^41^. Repetitive sequences were visualised with StainedGlass ^42^ and tandem repeats were annotated with ULTRA ^43^. Table 4 contains a list of all software tool versions used, where appropriate.

**Table 4.** Software tools used.

Software tool	Version	Source
hifiasm	0.14	28
purge_dups	1.2.3	29
SALSA2	2.2-4c80ac1	32
Long Ranger align	2.2.2	44
FreeBayes	1.3.1	30
MitoHiFi	2	37
gEVAL	N/A	34
HiGlass	1.11.6	35
PretextView	0.1.x	36
BlobToolKit	3.4.0	39
BUSCO	5.3.2	24
D-Genies	1.4	40
minimap2	2.24	41
StainedGlass	0.5	42
ULTRA	1.0.0 beta	43

## Ethics/compliance issues

The genetic resources accessed and utilised under this project were done so in accordance with the UK ABS legislation (Nagoya Protocol (Compliance) (Amendment) (EU Exit) Regulations 2018 (SI 2018/1393)) and the national ABS legislation within the country of origin, where applicable.

## Data Availability

European Nucleotide Archive: *Anopheles marshallii* genome assembly, idAnoMarsDA_429_01. Accession number PRJEB53264; https://identifiers.org/bioproject/PRJEB53264. The genome sequence is released openly for reuse. The *Anopheles marshallii* genome sequencing initiative is part of the Anopheles Reference Genomes project PRJEB51690. All raw sequence data and the assembly have been deposited in INSDC databases. Raw data and assembly accession identifiers are reported in Table 1. Members of Wellcome Sanger Institute Scientific Operations: Sequencing Operations are listed here: https://doi.org/10.5281/zenodo.12165051.
