# Supplementary material for: A chromosomal reference genome sequence for the malaria mosquito, Anopheles marshallii, Theobald, 1903
Source: Wellcome Open Res. 2024 Sep 26;9:554. [Version 1] doi: 10.12688/wellcomeopenres.22989.1 (PMC11538599; doi:10.12688/wellcomeopenres.22989.1)
Supplement: Peer review history

# Reviewer report: Reviewer response for version 1

**DOI:** 10.21956/wellcomeopenres.25314.r106640

**Published:** 2024-11-08

**Authors:** Din Syafruddin

**Affiliations:** 1 Hasanuddin University Medical Research Center (HUMRC), National Research and Innovation Agency (BRIN), Makassar, Indonesia

**General comment:**

 The authors presented a complete genome sequence of *Anopheles marshalli*, including both chromosomal and extrachromosomal (mitochondrial) genomes. Therefore, the title should represent the entire results and should be changed accordingly. Complete genome sequence of many major malaria vectors such as *Anopheles gambiae* and *An. dirus* have been available in the GenBank and the authors could compare their results with the 2 malaria mosquitoes.

**Suggested Title:**

 A reference genome sequence for the malaria mosquito, *Anopheles marshallii*, Theobald, 1903

**Specific comment:**

1. The authors reported the complete sequence of mitochondrial DNA. “ *The complete mitochondrial genome was also assembled and is 15.4 kilobases in length”.  Th*e finding should also be discussed and compared with the previously reported mitochondrial genomes of other *Anopheles* species, such as An. gambiae etc. Otherwise, it may be published separately.
2. The authors may also discussed differences of *Anopheles marshalli* with the major malaria vector in Africa, *Anopheles gambiae*

**Recommendation: **Revise

Are sufficient details of methods and materials provided to allow replication by others?

Yes

Is the rationale for creating the dataset(s) clearly described?

Yes

Are the datasets clearly presented in a useable and accessible format?

Yes

Are the protocols appropriate and is the work technically sound?

Yes

Reviewer Expertise:

NA

I confirm that I have read this submission and believe that I have an appropriate level of expertise to confirm that it is of an acceptable scientific standard, however I have significant reservations, as outlined above.

# Reviewer report: Reviewer response for version 1

**DOI:** 10.21956/wellcomeopenres.25314.r103927

**Published:** 2024-11-06

**Authors:** Craig S Wilding

**Affiliations:** 1 Liverpool John Moores University, Liverpool, UK

This Genome Note provides a useful review of the genome assembly and annotation efforts for *Anopheles marshalli*, the first genome from the Marshallii group and therefore an important reference genome for further research in this group.

**Background:**

 It would be good to see some wider taxonomic context – approximately when did marshallii and the gambie complex last share a common ancestor (especially given the later dot plot). But this would be also useful to know given they are likely to be found sympatrically.

 These are minor pedantic points, but I would suggest two changes:

 Rather than "that inhabits across sub-Saharan Africa" I suggest " is found across sub-Saharan Africa"

 I would suggest changing "its generalist, including feeding on humans, behaviour" to "its generalist behaviour, including feeding on humans,"

**Methods:**

 Where the specimen is referred to as idAnoMarsDA-429_01 should this be ID AnoMarsDA-429_01?

 Given the projected closure of Vectorbase (https://www.nature.com/articles/d41586-024-03196-6) albeit with a temporary reprieve, it seems advisable to not suggest that the annotation will be made available there. All DToL assemblies/annotations are made available on Ensembl! so I would argue that this sentence is unnecessary.

**Results:**

 Figure 2 shows a blob plot. However, there is no mention of the significance of the small blobs outside the main area. What are these? From symbionts? This should at least be discussed.

 Figure 4. I am really unsure what this is showing me and what the point of it is. It seems to have been done because it could be done, rather than because it needed doing. Whilst there is mention of it showing synteny of predicted centromere locations with those of *An. gambiae* it is really not clear to me how it shows this. It could be better explained or the figure annotated to indicate this. It would also be useful to have some context about how closely related *An. marshallii *and *A. gambiae* are.

 There is no mention of the size and completeness of the mitochondrial genome.

Are sufficient details of methods and materials provided to allow replication by others?

Yes

Is the rationale for creating the dataset(s) clearly described?

Yes

Are the datasets clearly presented in a useable and accessible format?

Yes

Are the protocols appropriate and is the work technically sound?

Yes

Reviewer Expertise:

NA

I confirm that I have read this submission and believe that I have an appropriate level of expertise to confirm that it is of an acceptable scientific standard.

# Reviewer report: Reviewer response for version 1

**DOI:** 10.21956/wellcomeopenres.25314.r106646

**Published:** 2024-11-05

**Authors:** Mary E. Gebhardt, Renee Ali

**Affiliations:** 1 Molecular Microbiology and Immunology, Johns Hopkins University Bloomberg School of Public Health, Baltimore, Maryland, USA

In this article, the authors present a whole genome assembly of *Anopheles marshallii*, which is a secondary vector involved in malaria transmission. They state the known bionomic and behavioral knowledge of this species and outline the importance of contributing comprehensive genetic data to help further our knowledge of population genetics and phylogenetic studies among secondary vector species. The authors then summarize their methods for extraction, sequencing, and annotation of a single female *An. marshallii* from Gabon. 

 The protocols used in this article are sound and easy to follow and the data is publicly available and easy to access. There are a few small clarifications that could be made to enhance the article:

1. How was this species verified as *An. marshallii*? Were the specimens used morphologically verified by an expert? Some publicly available genetic data can be mislabeled without correct and verified morphology. 
2. For the annotation, why were separate non-related mosquitoes used instead of the parent or another sibling?

Are sufficient details of methods and materials provided to allow replication by others?

Yes

Is the rationale for creating the dataset(s) clearly described?

Yes

Are the datasets clearly presented in a useable and accessible format?

Yes

Are the protocols appropriate and is the work technically sound?

Yes

Reviewer Expertise:

Anopheles ecology and genetics

We confirm that we have read this submission and believe that we have an appropriate level of expertise to confirm that it is of an acceptable scientific standard.

# Reviewer report: Reviewer response for version 1

**DOI:** 10.21956/wellcomeopenres.25314.r106648

**Published:** 2024-11-02

**Authors:** Abdou Azaque Zouré

**Affiliations:** 1 University of Liège, Institute of Health Sciences Research, (IRSS/CNRST), Gembloux, Belgium

- I propose to make the link with the *An.gambiae *complex, which is the main malaria vector.
- I will then propose a phylogenetic tree that puts together the sequences of *An gambiae*? your *Anopheles marshallii* and if possible other *Anopheles marshallii* sequences.
- In the methods, you need to give a lot of information about the collection site.

Are sufficient details of methods and materials provided to allow replication by others?

Yes

Is the rationale for creating the dataset(s) clearly described?

Yes

Are the datasets clearly presented in a useable and accessible format?

Yes

Are the protocols appropriate and is the work technically sound?

Yes

Reviewer Expertise:

Genetic, molecular biology, genomic

I confirm that I have read this submission and believe that I have an appropriate level of expertise to confirm that it is of an acceptable scientific standard.
